# Differential weighting of temporal envelope cues from the low-frequency region for Mandarin sentence recognition in noise

**DOI:** 10.1186/s12868-022-00721-z

**Published:** 2022-06-13

**Authors:** Yang Guo, Zhong Zheng, Keyi Li, Yuanyuan Sun, Liang Xia, Di Qian, Yanmei Feng

**Affiliations:** 1 Department of Otolaryngology-Head and Neck Surgery, Shanghai Jiao Tong University Affiliated Sixth People’s Hospital, 600 Yishan Road, Xuhui District, Shanghai, 200233 China; 2 Department of Otorhinolaryngology Head and Neck Surgery, Shanghai Key Clinical Disciplines of Otorhinolaryngology, Eye and ENT Hospital of Fudan University, 83 Fenyang Road, Xuhui District, Shanghai, 200031 China; 3 Sydney Institute of Language & Commerce, Shanghai University, 20 Chengzhong Road, Jiading District, Shanghai, 201800 China; 4 Department of ENT, People’s Hospital of Shenzhen Longhua District, 38 Jianshe-East Road, Longhua District, Shenzhen, 518109 China

**Keywords:** Sentence recognition, Relative weight, Temporal envelope cues, Noise, Mandarin Chinese, Cochlear implants

## Abstract

**Background:**

Temporal envelope cues are conveyed by cochlear implants (CIs) to hearing loss patients to restore hearing. Although CIs could enable users to communicate in clear listening environments, noisy environments still pose a problem. To improve speech-processing strategies used in Chinese CIs, we explored the relative contributions made by the temporal envelope in various frequency regions, as relevant to Mandarin sentence recognition in noise.

**Methods:**

Original speech material from the Mandarin version of the Hearing in Noise Test (MHINT) was mixed with speech-shaped noise (SSN), sinusoidally amplitude-modulated speech-shaped noise (SAM SSN), and sinusoidally amplitude-modulated (SAM) white noise (4 Hz) at a + 5 dB signal-to-noise ratio, respectively. Envelope information of the noise-corrupted speech material was extracted from 30 contiguous bands that were allocated to five frequency regions. The intelligibility of the noise-corrupted speech material (temporal cues from one or two regions were removed) was measured to estimate the relative weights of temporal envelope cues from the five frequency regions.

**Results:**

In SSN, the mean weights of Regions 1–5 were 0.34, 0.19, 0.20, 0.16, and 0.11, respectively; in SAM SSN, the mean weights of Regions 1–5 were 0.34, 0.17, 0.24, 0.14, and 0.11, respectively; and in SAM white noise, the mean weights of Regions 1–5 were 0.46, 0.24, 0.22, 0.06, and 0.02, respectively.

**Conclusions:**

The results suggest that the temporal envelope in the low-frequency region transmits the greatest amount of information in terms of Mandarin sentence recognition for three types of noise, which differed from the perception strategy employed in clear listening environments.

**Supplementary Information:**

The online version contains supplementary material available at 10.1186/s12868-022-00721-z.

## Introduction

By 2050, hearing loss is expected to affect 900 million people worldwide [1]. The cochlear implant (CI) is one of the most successful prostheses [2]. To date, more than 700,000 patients globally have been fitted with Cis; most of these patients communicate fluently under in clear conditions [3–5]. Although CIs allow users to understand up to 90% of all words in sentences spoken in clear environments, further challenges are encountered in noisy environments [3]. In CIs, the only signals transmitted are the temporal envelope cues of various frequency regions; the temporal fine structure (TFS) cues of the original acoustic signals are discarded. Many scholars have suggested that the lack of TFS cues partly explains the hearing difficulties experienced in noisy environments [6–9].

China accounts for approximately 20% of the world’s population and the socioeconomic burdens of hearing loss in China are immense [10]. By a conservative extrapolation, there is an estimated annual demand of 100,000 CIs in China [11]. The widespread use of CIs, which transmit only temporal envelope cues, by Chinese speakers also sparks a theoretical interest in the contribution of temporal envelope cues across frequency regions to Mandarin perception, which, unlike English, is a tonal language. Therefore, in this study, we used temporal envelope cues under noisy conditions to focus on the perception strategies adopted by Chinese speakers for Mandarin perception. This was done with the ultimate goal of developing optimal CIs for Chinese-speaking CI users.

To simulate the stimulation pattern of CIs, Shannon et al. divided the frequency spectrum into continuous broad-frequency bands (i.e., analysis filters) and then extracted the temporal envelope cues from different frequency bands to modulate noises of the same bandwidths [12]. The recognition performance increased with the number of bands [12]. The number of frequency bands needed for good speech recognition increased with the increasing difficulty of the listening situation [13, 14]. Some researchers allocated different frequency bands to different frequency regions, each containing several continuous-frequency bands [12, 15–18]; they found that temporal cues delivered at various frequencies contribute unequally to speech intelligibility [16–25]. The different frequency regions were presented to listeners to acquire recognition accuracies. The relative weights of temporal cues from various frequency regions could be calculated by permutation and combination of temporal information in different frequency regions. In this study, the frequency-weighting function of the temporal envelope was used to indicate the relative weights of the temporal envelope in different frequency regions [21, 25, 26].

Ardoint et al. extracted temporal envelope cues from 15 frequency bands across 70–7313 Hz and divided them into five regions. Consonant identification scores were obtained by presenting normal-hearing listeners with envelope cues from a single region and pairs of regions under clear conditions. The results suggested that temporal envelopes in the high-frequency region (1.8–7.3 kHz) contributed more than those of other regions toward English consonant recognition under clear listening conditions [17]. In contrast, another “hole” method (i.e., spectral removal method [16]) was used to study the weighting function of the temporal envelope in various frequency regions. Shannon et al. eliminated the information in low-, middle-, or high-frequency regions to simulate holes in the apical, middle, or basal regions of the cochlea. Recognition results suggested that the hole in the apical region (i.e., loss of temporal envelope cues in the low-frequency region) was more damaging than holes in the middle or basal regions [16]. These conflicting observations might result from the different spectra, cutoff frequency allocations, and methods used for extracting the envelope. In addition, Shannon et al. only investigated the effect of a single hole in the spectrum, which did not take into account the synthetic effects of nonadjacent frequency regions, and the negative effect of the hole was not obvious when the size was relatively small [16].

Kasturi et al. modified the setting of hole conditions in their study, considering the possibility that listeners could combine speech cues from nonadjacent frequency regions [18]. The speech materials spanning the frequency range from 300 to 5500 Hz were filtered into six frequency regions in a logarithmic fashion. The hole in the frequency spectrum was created by removing the information cues in one or two frequency regions. The intelligibility of speech with a single hole in different regions, or with two holes in disjointed or adjacent regions in the spectrum, was assessed. Then, the intelligibility of speech without holes was obtained as a baseline. Then, the frequency-weighting functions were derived based on a least-squares approach, which suggested that all frequency ranges contributed equally to consonant identification, whereas frequency regions located at 300–487, 791–1284, and 1284–2085 Hz, received the largest weights for vowel identification [18].

In contrast to English, which is a non-tonal language, Mandarin Chinese is a tonal language. This means that lexical tones are critical and essential features of the language, and changing pitches are associated with different meanings [27, 28]. There are four distinctive tone patterns in Mandarin Chinese, and these are characterized by the syllable-level fundamental frequency (F0) contours: high tone (tone 1), rising tone (tone 2), dipping tone (tone 3), and falling tone (tone 4) [29]. For instance, the Mandarin Chinese syllable /ma/ has four different tones: mā (Tone 1, high, 55(the numbers represent tone height); e.g., “mother”), má (Tone 2, rising, 35; e.g., “hemp”), mă (Tone 3, dipping, 214; e.g., “horse”), and mà (Tone 4, falling, 51; e.g., “scold”). It is well acknowledged that lexical tone plays a major role in the understanding of Mandarin speech [30–33]. Fu et al. found that tone, vowel, and consonant recognition contributed equally to Chinese sentence recognition [27]. Incorrect tone negatively influenced Mandarin sentence recognition in ways similar to misplaced or missing consonants and vowels in sentences [31, 33].

Recently, we studied the frequency-weighting functions of temporal envelope cues for Mandarin sentence recognition in a clear environment [25]. The temporal envelope cues of the original sentences were extracted across 80–7562 Hz and then distributed into five spectral frequency regions. The relative temporal envelope weights of the different regions were calculated after measuring the recognition scores under various conditions with different combinations of envelopes in different frequency regions. We found that temporal envelope cues in Region 1 (80–502 Hz) were of higher weight than those in any other region for Mandarin sentence perception [25], which differs from English speakers. This may be because Mandarin is a tonal language with different tones that convey different meanings [25]. Lexical tone recognition is crucial to Mandarin sentence perception and the role of F0 is essential in tone perception. Therefore, it is logical that Region 1 should exhibit a high relative weight in terms of Mandarin sentence perception [34–37]. However, the perceptual weighting strategy may differ depending on the listening environment.

Under clear listening conditions, the acoustic cues of speech are typically abundant and conducive to successful recognition. However, CI users encounter difficulties under noisy conditions [3]; this is a problem because most conversations in the real world occur in noisy environments. Several studies have addressed the perceptual weight shifts of envelope cues across various frequency regions for English recognitions in noise. However, no research to date has focused on the change of perceptual weights for Chinese Mandarin in noisy environments.

Speech-shaped noise (SSN) that matches the long-term average spectrum of recorded speech material is frequently applied in tests investigating the relative weights of temporal cues from various frequency regions [9, 19, 38]. This ensures that the signal-to-noise ratios (SNRs) are approximately equal at all frequencies [39, 40]. Using both the hole method and correlational method [41], Apoux and Bacon studied the relative temporal envelope weights of four frequency regions in SSN [19]. Under clear listening conditions, the hole method showed that the temporal envelope cues of all regions contributed similarly to consonant identification. However, under noisy conditions, both the hole method and correlational method indicated that the temporal envelope cues in the highest frequency region had the greatest importance [19]. Although low-rate syllabic modulations (< 4 Hz) are present across the frequency spectrum, mid- and/or high-frequency modulations (> 10 Hz) might carry unique speech information specific to the high-frequency regions [9, 19]. The shapes of the modulation spectra in adjacent frequency regions might explain this weight shift observed by Apoux and Bacon [19].

In addition, most realistic noises are modulated or fluctuating in level; therefore, fluctuating background noises (i.e., amplitude-modulated noise) are widely used in perception experiments [15, 42–45]. Amplitude modulation was found to interfere with the perception of temporal envelope cues, especially with low modulation rates [15, 46]. Fogerty also found that listeners placed higher perceptional weight on temporal envelope cues in the high-frequency region if speech was interrupted by noise at either a syllabic rate (4 Hz) or periodic rate (128 Hz) [9]. Thus, listeners would adapt their perceptual strategies, namely frequency-weighting functions, when communicating in adverse environments (i.e., those with noise) [9, 38]. Although there were evidences that white noise could severely impair the speech perception [47, 48], there has been no study focusing on the impacts of white noise on the relative weights of the temporal envelope from different frequency regions.

Investigating the perception strategy using envelope cues has important implications because the number of CI users who speak Chinese is growing rapidly, and CIs primarily convey envelop cues. Taking into account that the tonal character of Mandarin and the essential roles of F0 in lexical tone recognition, it is expected that temporal envelope cues from the low-frequency region, where F0 (typically ranges approximately from 100 to 350 Hz for Mandarin lexical tones) falls in [32, 49, 50], are more important for Mandarin sentence recognition under noisy conditions than in clear listening conditions. Furthermore, it is hypothesized that the weights of low-frequency region would differ under various kinds of noises. In this study, we tested these hypotheses by changing the number and location of holes in the spectrum. Then, we adopted a least-squares approach to determine the relative weights of temporal envelope cues across frequency regions in different noisy environments.

## Methods

### Participants

A total of 40 participants were recruited and allocated into different test groups (see Table 1). All subjects were Shanghai Jiao Tong University graduate students who were native Mandarin speakers from different provinces in mainland China and fluent in their own local dialects. All listeners were not previously exposed to the test sentences. All subjects had audiometric pure-tone thresholds of ≤ 25 dB HL from 0.25 to 8 kHz. The study was approved by the Ethics Committee of Shanghai Jiao Tong University Affiliated Sixth People’s Hospital. All participants signed informed consent forms before testing and were compensated for their participation in the study.

**Table 1:** Assignment of conditions for different groups and numbers of subjects for cognition tests in SSN, SAM SSN, and SAM white noise

Background noises	SSN		SAM SSN		SAM white noise	
Group	Group 1	Group 2	Group 3	Group 4	Group 5	Group 6
Numbers of subjects	5 (3 males and 2 females)	5 (3 males and 2 females)	5 (2 males and 3 females)	5 (2 males and 3 females)	10 (5 males and 5 females)	10 (5 males and 5 females)
Conditions	Full region	Hole 1 + 3	Full Region	Hole 1 + 3	Full region	Hole 1 + 3
	Hole 1	Hole 1 + 4	Hole 1	Hole 1 + 4	Hole 1	Hole 1 + 4
	Hole 2	Hole 1 + 5	Hole 2	Hole 1 + 5	Hole 2	Hole 1 + 5
	Hole 3	Hole 2 + 4	Hole 3	Hole 2 + 4	Hole 3	Hole 2 + 4
	Hole 4	Hole 2 + 5	Hole 4	Hole 2 + 5	Hole 4	Hole 2 + 5
	Hole 5	Hole 3 + 5	Hole 5	Hole 3 + 5	Hole 5	Hole 3 + 5
	Hole 1 + 2	Hole 1 + 2	Hole 1 + 2	Hole 1 + 2	Hole 1 + 2	Hole 1 + 2
	Hole 2 + 3	Hole 2 + 3	Hole 2 + 3	Hole 2 + 3	Hole 2 + 3	Hole 2 + 3
	Hole 3 + 4	Hole 3 + 4	Hole 3 + 4	Hole 3 + 4	Hole 3 + 4	Hole 3 + 4
	Hole 4 + 5	Hole 4 + 5	Hole 4 + 5	Hole 4 + 5	Hole 4 + 5	Hole 4 + 5

Each test group contained 10 conditions corresponding to 10 randomly selected lists of Mandarin version of Hearing in Noise (MHINT) materials. The participants enrolled for testing in SSN ranged from 21 to 36 years old (average = 24.9); the participants enrolled for testing in SAM SSN ranged from 21 to 26 years old (average = 22.6); and the participants enrolled for testing in SAM white noise ranged from 20 to 27 years old (average = 23.8)

### Design

The sentence recognition scores were low (8–16% correct) when temporal envelope information from only one frequency region was delivered under clear listening conditions [25]. We hypothesized that the scores obtained when delivering temporal envelope information from only one frequency region in noise would be lower, which was too low to allow the relative weights of the temporal envelope values of different regions to be explored using the least-squares approach [18]. This was confirmed in a pilot experiment. Therefore, we adopted the hole method, which mimics dead regions or spectral holes in the cochlea [16, 51]. We employed five frequency regions and created different hole conditions. The current study consists of two parts: single-hole and two-hole sentence identification tasks. The MHINT sentences were used in the two recognition tasks. Five frequency regions (Table 2) were manipulated to create 16 frequency conditions for each sentence in the MHINT list.

**Table 2:** Cutoff frequencies for frequency regions of the temporal envelope

Frequency region	Lower frequency (Hz)	Upper frequency (Hz)
Region 1	80	502
Region 2	502	1022
Region 3	1022	1913
Region 4	1913	3856
Region 5	3856	7562

In a single-hole sentence recognition task, one baseline where all five frequency regions were presented was set as a control condition. The other nine experimental conditions (Table 1) were created by removing one or two frequency regions. Five single-hole conditions were made by removing only one frequency region. Correspondingly, the other four single-hole conditions were created by removing two adjacent frequency regions (e.g., Hole 1 + 2, Hole 2 + 3, etc.). Therefore, a total of 10 frequency conditions were employed in this task. For the nine experimental conditions, all removed frequency regions were filled with SSN, SAM SSN, or SAM white noise, respectively. In the two-hole sentence recognition task, four single-hole conditions were created by removing two adjacent frequency regions; this approach was identical to that of the single-hole sentence task. Furthermore, six two-hole conditions were created by removing two disjointed regions (e.g., Hole 1 + 3, Hole 2 + 4, etc.). As in the single-hole sentence identification task, all removed frequency regions were filled with SSN, SAM SSN, or SAM white noise introduced at an SNR of + 16 dB to prevent any possible use of information from the transitional bands [52, 53]. The filler noises were prepared using the same cutoff frequencies employed to prepare the frequency regions of the envelope cues. For simplicity, we abbreviated the various conditions. For example, “Hole 1” implies that the presented speech consisted of temporal envelope information from Frequency Regions 2–5 and a filler noise was used in Region 1. “Hole 1 + 2” refers to a speech stimulus consisting of envelope information from Frequency Regions 3–5 and filler noises from Regions 1 and 2. Finally, “Full Region” refers to a stimulus containing envelope information from all five frequency regions (i.e., Regions 1–5).

### Stimuli

The content of the speech material (i.e., MHINT) resembles everyday conversation, which is simple and can be easily understood by native Mandarin speakers with various levels of education and by children aged 6 and above [50]. The MHINT materials developed by Wong et al. contain only 12 lists, each with 20 sentences (e.g., “Dad brought home a watermelon today”; “Everybody likes to work together with him”; “I have a date tomorrow morning at 9 o'clock”; “The apples in the orchard are big and red”; and “There is a new classmate in our class”. The corresponding MHINT Chinese sentences were displayed in Additional file 1: Table S1) [50]. There are 10 key syllables in each sentence (Additional file 1: Table S1) [50]. The sentences in each list were equated for difficulty and distributions of phoneme and tone [50].

**Fig. 1:**
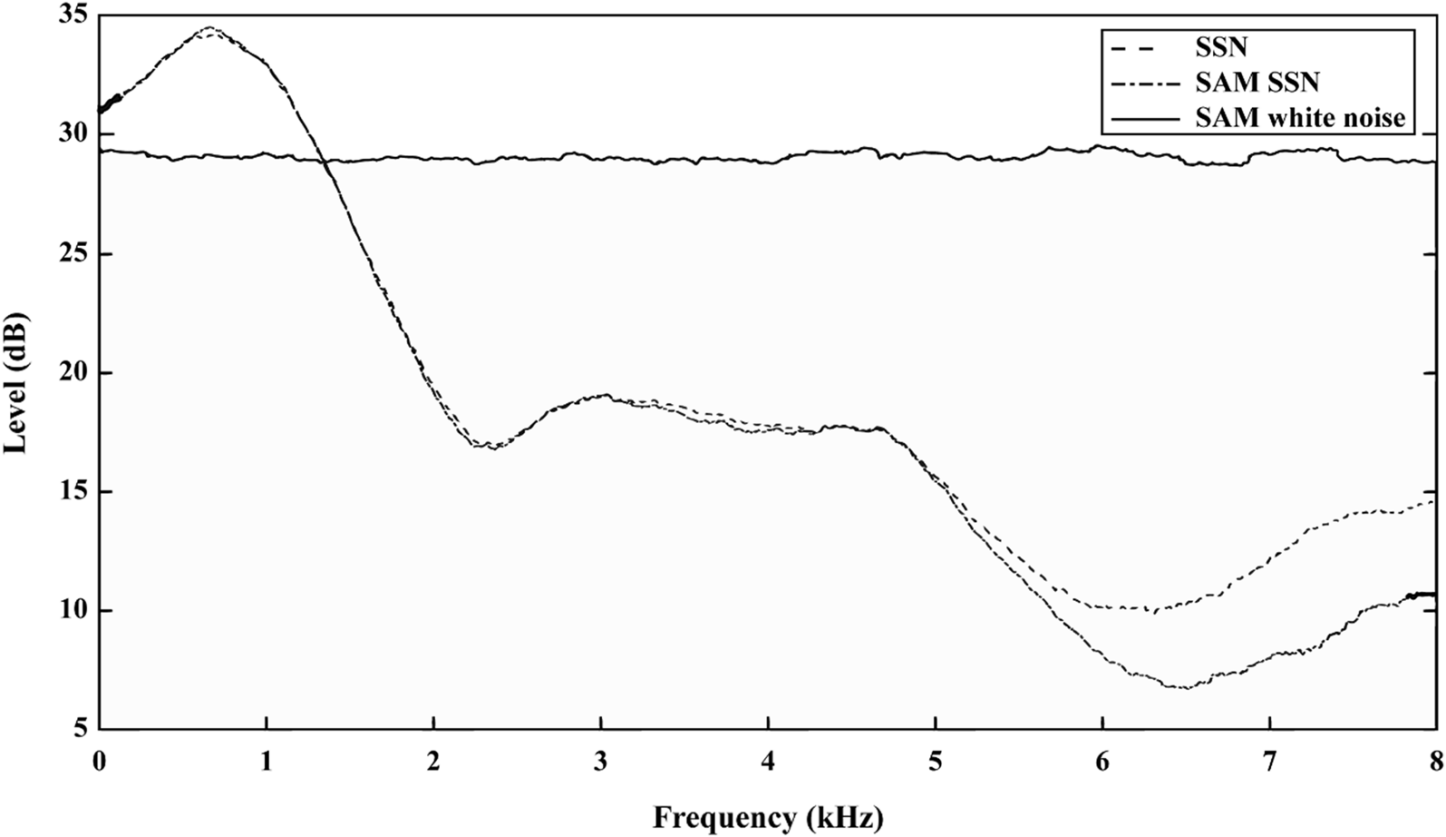
Spectra of the SAM white noise (solid line), SSN (dashed line), and SAM SSN (dotted line) for MHINT

SSN was created to match the long-term average spectrum of the MHINT sentences to simulate a typical (noisy) listening environment. It was also used as a masker (Fig. 1) [50, 54–56]. In addition, we combined the original speech material with sinusoidally amplitude-modulated (SAM) SSN. As demonstrated previously, a low-rate interruption of speech cues can affect perception [15], so we chose the modulation rate used by Fogerty (4 Hz) [9]. Thus, the SAM SSN refers to SSN modulated with a sinusoid of 4 Hz (100% depth). In addition, white noise modulated with a sinusoid of 4 Hz (100% depth) was also applied and is referred to as SAM white noise. The phase at which modulation commenced was randomized across sentences. During our pilot experiment, we found that the SNR of + 5 dB could result in recognition scores varying from about 15 to 95% under different conditions. Thus, the SNR was set at + 5 dB. The SSN, SAM SSN, or SAM white noise started 500 ms before the target sentence commenced and ended 500 ms after the end of the sentence.

The speech material/noise mixture was initially filtered into 30 adjacent, logarithmically spaced frequency bands spanning from 80 to 7562 Hz using zero-phase, third-order Butterworth filters (18 dB/oct slopes). Therefore, the width of each band was one equivalent rectangular bandwidth (ERB_N_) [57]. The temporal envelope of the signal was extracted from each band using the Hilbert transformation and then low-pass filtered at a cutoff of 64 Hz using a third-order Butterworth filter. Next, the amplitude of the white noise (the carrier) was modulated by the envelope. Then, the envelope-modulated noise from each band was band-limited using the same band-pass filters. The modulated noise bands were allocated to five frequency regions and presented to listeners under different conditions. The frequency-region assignments of the temporal envelope cues were identical to those of Guo et al. [25]. The cutoff frequencies of the various regions are listed in Table 2.

In accordance with the recognition conditions in Table 1, each group required 10 lists of MHINT materials for each listening background noise. For each group, 10 lists of MHINT sentences were randomly selected and assigned to each condition in this group, and the remaining two MHINT lists were used for practice. For example, for Group 3 under SAM SSN, Lists 2 –11 were selected and assigned to the 10 conditions, respectively. The remaining lists (List 1 and 12) were assigned as practice materials. For Group 5 under SAM white noise, MHINT sentence Lists 1–10 were selected and assigned to the 10 conditions, respectively. The remaining lists (List 11 and 12) were assigned as practice materials.

### Procedure

The experiment was performed in a sound-attenuating booth. Stimuli were delivered bilaterally at a comfortable listening level for each subject, usually 65 dB SPL, through Sennheiser HD 205 II circumaural headphones. Before formal testing, each subject attended a practice session. Feedbacks were provided during practice to familiarize the subjects with the stimuli. The practice continued until the subject’s performance plateaued (e.g., the number of correctly recognized words in different practice sentences under one specific condition did not change by more than two). If not, the subject would be asked to rest and participate in the experiments on another day. On that day, the subject’s performance should also reach a plateau before the formal test.

In the formal tests, every subject participated in a total of 10 testing conditions. The order of presentation of these testing conditions, corresponding to 10 lists of MHINT sentences, was randomized across the subjects. Although the training could acquaint the subjects with the distorted test stimuli, they could listen to a sentence as many times as desired before moving on to the next sentence [28]. This was to minimize the impact of the sentence distortions on the subjects. No feedback was provided during formal tests. Approximately 30% of the subjects required repetition of test sentences that were relatively difficult and confusing to recognize. This accounted for about 5% of the test sentences. Among this 30%, most subjects’ recognition results at the first listen were identical to those after repeated listening. All subjects were asked to repeat the syllables in the test sentences as precisely as possible and were permitted to guess if they were unsure. The subjects were instructed to immediately repeat the syllables they thought they heard during the test process, irrespective of whether the sentence was played over or not. The responses were recorded by the tester using pen and paper, and the scores were calculated by an independent researcher. Each key syllable in a sentence was scored as correct or incorrect. After one complete list of sentences was presented for one listening condition, the total number of correct syllables were counted and divided by the total number of syllables (i.e., 200). This resulted in the recognition scores for this condition. All subjects were allowed to take breaks whenever required. The results were analyzed using SPSS 22.0 software.

## Results

### Recognition scores under SSN

**Fig. 2:**
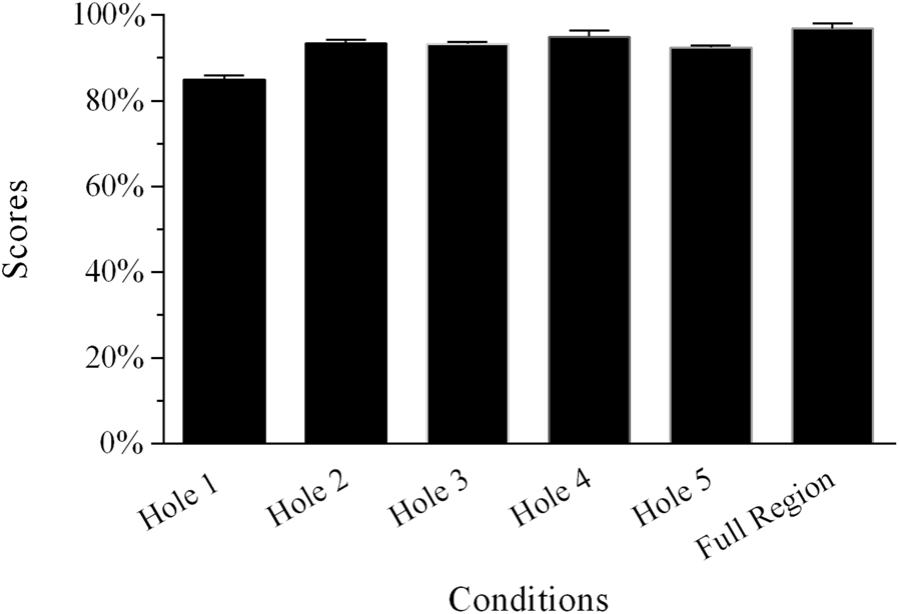
Mean percent-correct scores for Mandarin sentence recognition as a function of conditions without temporal envelope cues from one frequency region and the condition with all frequency regions for Group 1 using SSN. The error bars indicate standard errors

**Fig. 3:**
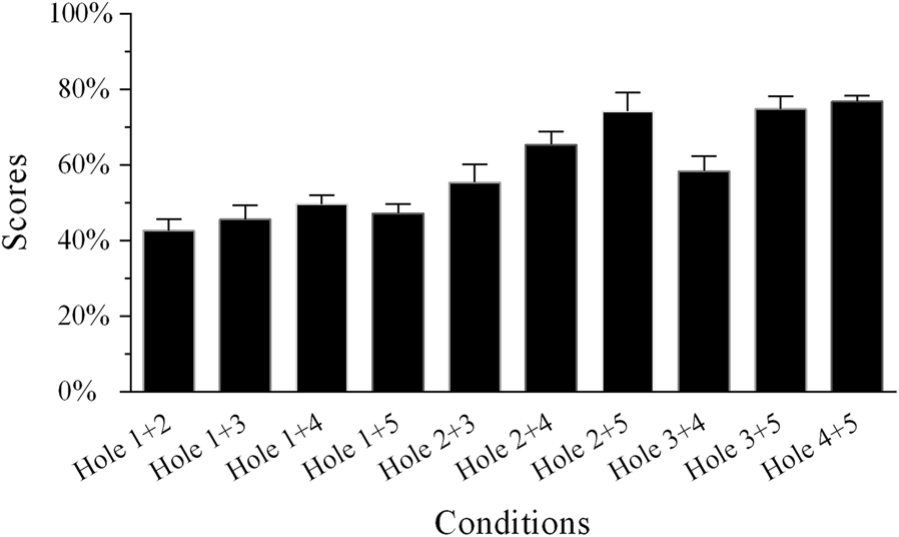
Mean percent-correct scores for Mandarin sentence recognition as a function of conditions without temporal envelope cues from two frequency regions for Group 2 using SSN. The error bars indicate standard errors

In the SSN tests, the recognition scores changed for different conditions (Figs. 2 and 3; Additional file 1: Table S2). The conditions for which temporal envelope cues were missing from two adjacent frequency regions were identical when using SSN for Groups 1 and 2. The independent samples *t*-test indicated that the percentage-correct scores of single holes created by removing two adjacent frequency regions in the two groups did not significantly differ from each other (all *p* > 0.05; Table 3). Thus, the data from the two groups were merged to explore the relative weights of frequency regions for MHINT sentence recognition in SSN. In the single-hole sentence-recognition task, when the temporal envelope from one frequency region was absent, the average percentage-correct sentence-recognition scores were lowest for Hole 1 and highest for Hole 4. The average score for the “Full Region” condition in SSN was 96.8%. After the rationalized arcsine units (RAU) transformation, a one-way repeated-measures analysis of variance (ANOVA) revealed that speech recognition scores significantly differed across the six conditions shown in Fig. 2 [*F*(5,20) = 22.543, *p* < 0.0001]. This was done using the frequency region condition with six levels as a within-subjects factor. Post hoc analysis with Bonferroni correction suggested that the scores for the Hole 1 condition were significantly lower than those for the other five conditions under SSN. The mean of the Full Region scores was the highest; it did not differ significantly from the other four conditions (i.e., Holes 2–5) under SSN.

**Table 3:** Comparison of percent-correct scores in SSN for conditions without temporal envelope cues from two adjacent frequency regions for the two groups

Conditions	Group 1	Group 2	*t*-test (*p* value)
Hole 1 + 2	47.7 ± 6.8 (%)	42.5 ± 6.9 (%)	0.266
Hole 2 + 3	60.5 ± 14.1 (%)	55.2 ± 10.5 (%)	0.521
Hole 3 + 4	66.7 ± 6.1 (%)	58.0 ± 8.7 (%)	0.106
Hole 4 + 5	72.4 ± 5.0 (%)	76.4 ± 3.2 (%)	0.174

In the two-hole sentence-recognition task, recognition scores under SSN when temporal envelope cues were lacking in two frequency regions are presented in Fig. 3. The lowest was associated with the Hole 1 + 2 condition and the highest with the Hole 4 + 5 condition. The results were subjected to one-way repeated measures ANOVA using the frequency region condition with 10 levels as the within-subjects factor. This showed a significant main effect of the different conditions on sentence recognition [*F*(9,36) = 13.839, *p* < 0.001]. Post hoc analysis verified this assumption, showing that the scores of the Hole 1 + 4 condition were significantly lower than those of the Hole 2 + 4 and Hole 4 + 5 conditions. In addition, the analysis suggested that the Hole 1 + 5 condition scores were significantly lower than those of the Hole 2 + 5, Hole 3 + 5, and Hole 4 + 5 conditions.

### Recognition scores under SAM SSN

**Fig. 4:**
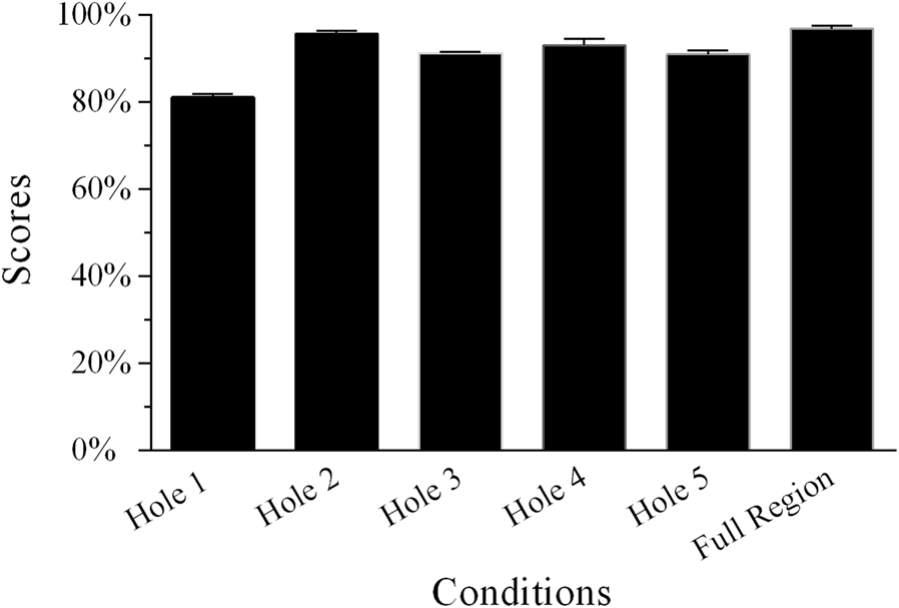
Mean percent-correct scores for Mandarin sentence recognition as a function of conditions without temporal envelope cues from one frequency region and the condition with all frequency regions for Group 3 using SAM SSN. The error bars indicate standard errors

**Fig. 5:**
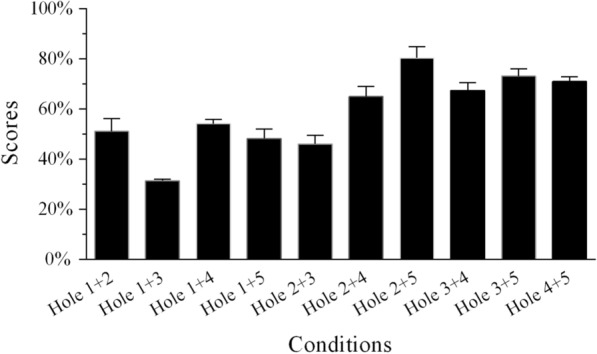
Mean percent-correct scores for Mandarin sentence recognition as a function of conditions without temporal envelope cues from two frequency regions for Group 4 using SAM SSN. The error bars indicate standard errors

In the SAM SSN tests, the recognition scores varied for different conditions (Figs. 4 and 5; Additional file 1: Table S3). As described above, the conditions in which temporal envelope cues were missing from two adjacent frequency regions were also identical when using SAM SSN for Groups 3 and 4. The independent samples *t*-test indicated that the percentage-correct scores of single holes created by removing two adjacent frequency regions in the two groups did not significantly differ from each other (all *p* > 0.05; Table 4). The data from the two groups were merged to explore the relative weights of frequency regions for MHINT sentence recognition in SAM SSN. In the single-hole sentence-recognition task, when the temporal envelope from one frequency region was absent, the average percentage-correct sentence-recognition score was lowest for Hole 1 and highest for Hole 2. The average score for the Full Region condition in SAM SSN was 96.8%. After RAU transformation, using the frequency-region condition with six levels as a within-subjects factor, a one-way repeated-measures ANOVA revealed that speech recognition scores significantly differed across the six conditions shown in Fig. 4 [*F*(5,20) = 71.585, *p* < 0.0001]. Post hoc analysis with Bonferroni correction suggested that the scores of the Hole 1 condition were significantly lower than those of the other five conditions under SAM SSN. The mean of the Full Region scores was the highest and did not differ significantly from those of the Hole 2, 3, and 4 conditions under SAM SSN. However, the scores of the Hole 5 condition were significantly lower than those of the Full Region and Hole 2 conditions under SAM SSN.

**Table 4:** Comparison of percent-correct scores in SAM SSN for conditions without temporal envelope cues from two adjacent frequency regions for the two groups

Conditions	Group 3	Group 4	*t*-test (*p* value)
Hole 1 + 2	43.3 ± 12.8 (%)	51.1 ± 11.4 (%)	0.340
Hole 2 + 3	36.2 ± 9.1 (%)	46.0 ± 7.9 (%)	0.106
Hole 3 + 4	62.8 ± 11.6 (%)	67.4 ± 6.8 (%)	0.467
Hole 4 + 5	68.2 ± 11.0 (%)	71.0 ± 4.1 (%)	0.606

In the two-hole sentence-recognition task, the recognition scores using SAM SSN when the temporal envelope cues were lacking in two frequency regions are presented in Fig. 5. The scores obtained for the Hole 1 + 3 condition were lowest, whereas those with the Hole 2 + 5 condition were highest. The results were subjected to one-way repeated measures ANOVA using frequency-region condition with 10 levels as the within-subjects factor, which showed a significant main effect of the different conditions on sentence recognition [*F*(9,36) = 26.846, *p* < 0.001]. Post hoc analysis verified this assumption, showing that the scores of the Hole 1 + 3 condition were significantly lower than those of the Hole 1 + 4 condition in SAM SSN. In addition, the scores of the Hole 2 + 3 condition were significantly lower than those of the Hole 2 + 5 condition in SAM SSN.

### Recognition scores under SAM white noise

**Fig. 6:**
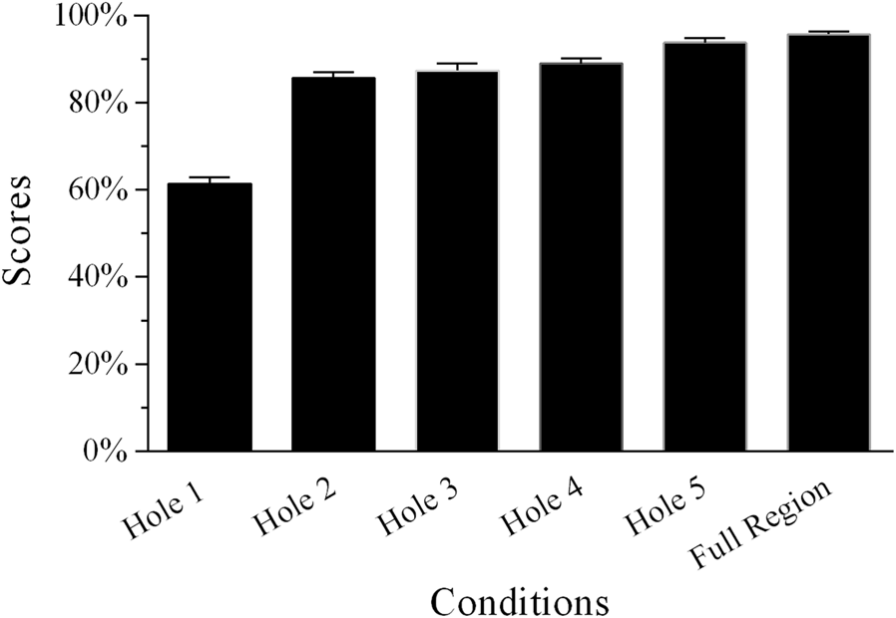
Mean percent-correct scores for Mandarin sentence recognition in SAM white noise as a function of conditions without temporal envelope cues from one frequency region and the condition with all frequency regions for Group 5. The error bars indicate standard errors

The conditions lacking temporal envelope cues from two adjacent frequency regions in the SAM white noise were identical for Groups 5 and 6. The independent samples *t*-test showed that the differences of the percentage-correct scores of single holes created by removing two adjacent frequency regions in the two groups were insignificant (all *p* > 0.05; Table 5). Therefore, we aggregated the data from the two groups to explore the relative weights of the five frequency regions. In the single-hole sentence-recognition task, when the temporal envelope from one frequency region was lacking, scores increased from Hole 1 to Hole 5 in SAM white noise (Fig. 6; Additional file 1: Table S4). The average score for the Full Region condition was 95.7% correct. Statistical significance was determined by using the percent-correct score as the dependent variable and the frequency region condition with six levels as the within-subjects factor. The scores were transformed to RAUs prior to statistical analyses to avoid probable ceiling or floor effects [58]. One-way repeated-measures ANOVA revealed that speech recognition scores significantly differed across the six conditions shown in Fig. 6 [*F*(5,45) = 118.977, *p* < 0.001]. Post-hoc analysis with Bonferroni correction revealed that the scores for Hole 1 were significantly lower than those for the other five conditions. The scores for Hole 5 and Full Region were similar and significantly higher than those of the other four conditions.

**Table 5:** Comparison of percent-correct scores in SAM white noise for conditions without temporal envelope cues from two adjacent frequency regions for the two groups

Conditions	Group 5	Group 6	*t*-test (*p* value)
Hole 1 + 2	22.3 ± 3.1 (%)	23.8 ± 5.3 (%)	0.436
Hole 2 + 3	18.3 ± 4.6 (%)	18.4 ± 5.8 (%)	0.967
Hole 3 + 4	68.2 ± 7.6 (%)	69.5 ± 6.8 (%)	0.692
Hole 4 + 5	84.1 ± 4.3 (%)	84.7 ± 4.2 (%)	0.735

**Fig. 7:**
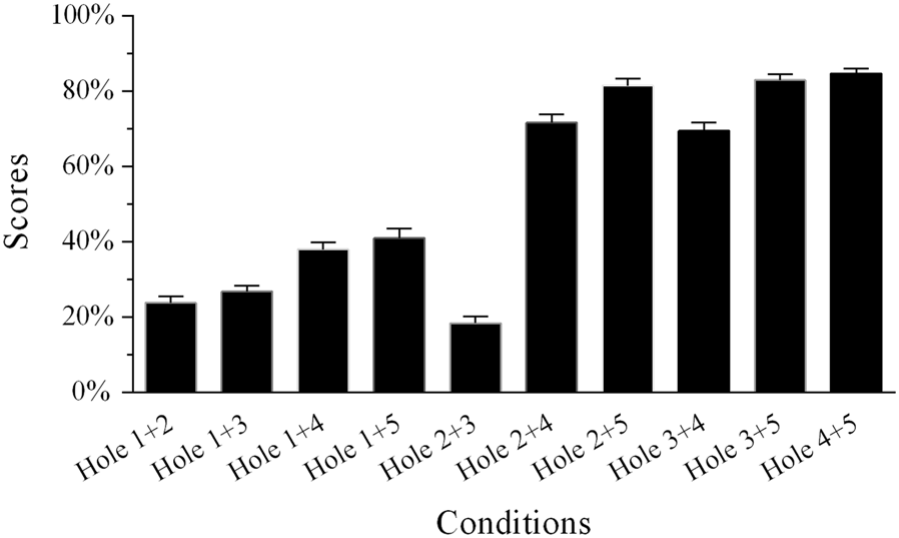
Mean percent-correct scores for Mandarin sentence recognition in SAM white noise as a function of conditions without temporal envelope cues from two frequency regions for Group 6. The error bars indicate standard errors

Figure 7 shows the sentence recognition scores obtained under various conditions when the temporal envelope cues from two frequency regions were absent in the two-hole sentence recognition task. The scores varied widely across the different conditions; the lowest was associated with the Hole 2 + 3 condition and the highest with the Hole 4 + 5 condition. Statistical significance was determined using the frequency region condition with 10 levels as a within-subjects factor. A one-way repeated-measures ANOVA revealed a significant main effect of conditions on sentence recognition [*F*(9,81) = 342.389, *p* < 0.001]. In general, the scores seemed to increase as the distance between the two absent frequency regions increased. Post-hoc analysis with Bonferroni correction verified this assumption, showing that the sentence recognition scores of the Hole 1 + 2 and Hole 1 + 3 conditions were significantly lower than those of Hole 1 + 4 and Hole 1 + 5 conditions. The sentence recognition scores of the Hole 2 + 3 condition were significantly lower than those of the Hole 2 + 4 and Hole 2 + 5 conditions. The sentence recognition scores of the Hole 3 + 4 condition were significantly lower than those of Hole 3 + 5 condition.

### Relative weights of temporal envelope cues from five frequency regions under different noisy conditions

The least-squares approach proposed by Kasturi et al. [18] was used to investigate the relative weights of the different frequency regions in terms of Mandarin sentence recognition under noisy conditions employing the temporal envelope. A linear combination of the strength of each frequency region was used to predict responses. 0 or 1 was employed to indicate whether the region was absent or present, respectively. Then, we minimized the sum of all squared prediction errors to obtain raw weights for all regions, which were then normalized for every subject such that the sum of the weights of the five frequency regions was unity.

**Fig. 8:**
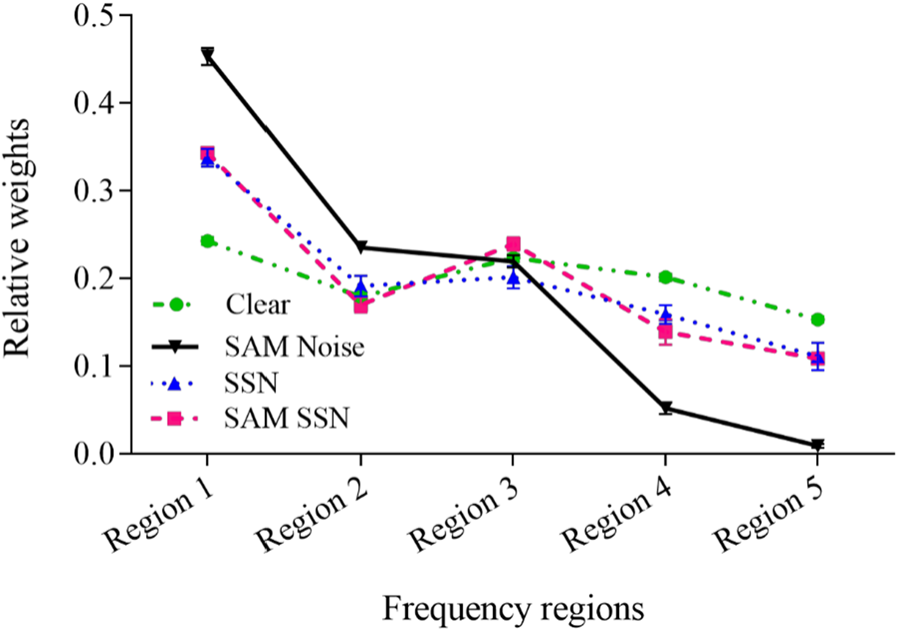
Relative weights of different frequency regions for Mandarin sentence recognition using temporal envelope cues under clear listening conditions and different background noises. The error bars indicate standard errors. The relative weight data for the clear listening conditions was adopted from Guo et al. [25]

The mean relative weights of Regions 1–5 for the listeners in a clear listening background and in the presence of SAM white noise, SSN, and SAM SSN are shown in Fig. 8 and Table 6. A two-way ANOVA conducted on the Regions (Region 1–5) and listening backgrounds (clear listening background, SSN, SAM SSN, and SAM white noise) indicated a significant effect of the Regions [*F*(4,180) = 626.0, *p* < 0.0001, *η*^*2*^ = 0.933] and a significant interaction between the Regions and listening backgrounds [*F*(12,180) = 122.5, *p* < 0.0001, *η*^*2*^ = 0.891]. However, there was no significant effect of the listening backgrounds [*F*(3,180) = 1.140, *p* = 0.334,, *η*^*2*^ = 0.019], as the sum of the weights across the frequency regions was 1 for all listening backgrounds.

**Table 6:** Comparison of mean relative weights of temporal envelope in clear environment and noises for different frequency regions

Frequency regions	Clear	SAM white noise	SSN	SAM SSN
1	0.25^a^	0.46^a,b^	0.34^a,b^	0.34^a,b^
2	0.18	0.24^b^	0.19	0.17
3	0.22	0.22	0.20	0.24
4	0.20	0.06^b^	0.16^b^	0.14^b^
5	0.15	0.02^b^	0.11^b^	0.11^b^

^a^The relative weight of Region 1 was significantly higher than those of other Regions in certain listening environments

^b^The difference in relative weights between this noise condition and the clear listening condition was statistically significant. The relative weight data for clear listening conditions was adopted from Guo et al [25]

Considering that the interaction between the Regions and listening background was significant, a simple effect analysis with Bonferroni correction were used to compare the mean weights for each Region under various listening conditions. Under clear listening condition, relative weights differed significantly between any two Regions. The relative weight of Region 1 was highest, followed by the weights of Region 3, 4, 2 and 5, successively. For SSN, the relative weight of Region 1 was highest, and the relative weight of Region 5 was lowest. The relative weight of Region 3 was significantly higher than that of Region 4, although the relative weight of Region 2 presented no significant difference compared with the weights of Regions 3 and 4. For SAM SSN, the relative weights of Regions 1 and 3 were higher than the weights of other Regions, whereas the relative weight of Region 1 was higher than that of Region 3. The relative weight of Region 2 was significantly higher than that of Region 5, although the relative weight of Region 4 presented no significant difference compared with the weights of Regions 2 and 5. For SAM white noise, the relative weight of Region 1 was highest among all Regions, and the relative weights differed significantly between any two frequency regions, except between Regions 2 and 3.

Besides, a simple effect analysis with Bonferroni tests showed that the relative weight of Region 1 for SAM white noise was highest among the four backgrounds, and the relative weights of Region 1 for SAM SSN and SSN were significantly higher than that in the clear listening condition. For Region 2, Bonferroni tests indicated that the relative weight for SAM white noise was higher than those of the other three conditions. For Region 3, Bonferroni tests indicated that the relative weight for SAM SSN was higher than that of the SSN condition. For Regions 4 and 5, Bonferroni tests showed that the relative weights of the two regions in SAM white noise were the lowest among the four conditions, and the relative weights for SAM SSN and SSN were significantly lower than that in the clear listening condition. The relative weights for the two regions in SAM SSN and SSN did not differ from each other. Overall, Region 1 was the most weighted among the five Regions in all three kinds of noise, and the relative weights for Region 1 in three kinds of noise were all significantly higher than that in the clear listening background.

## Discussion

In this study, we determined the relative contributions made by temporal envelope cues across different frequency regions for Mandarin sentence recognition in noisy backgrounds. More weights were placed in the low-frequency region, when the frequency-weighting functions of the temporal envelope for recognizing Mandarin sentences in noisy conditions were compared to those in clear listening conditions. There are several possible explanations for this finding.

First, Mandarin is a tonal language; this means that the same phonemes spoken with different tones have different meanings [27, 28]. Of the cues conveying lexical information, F0 is the most important in terms of lexical recognition [34, 35]. Discarding F0 cues could reduce the lexical tone recognition performance to a level that was only slightly above chance (32.7%); this was much poorer than the recognition of natural speech [28]. Furthermore, the amplitude contour curves of temporal envelope cues co-vary with changes in F0 over time in Mandarin, and periodicity cues embedded in the temporal envelope cues are directly correlated with changes in F0 [36]. Luo and Fu [37] demonstrated that tone identification could be improved by modifying the overall amplitude contours with reference to the F0 contours. For Mandarin lexical tones, Region 1 (80–502 Hz) in our study covers the typical frequency ranges of F0, from 100 to 350 Hz. Given the crucial role played by lexical tone recognition in Mandarin sentence perception and F0 in tone perception, it is logical that Region 1 should exhibit a high relative weight in terms of Mandarin sentence perception.

Second, lexical tone recognition was more important for Mandarin sentence perception under noisy conditions than in clear conditions. Chen et al. [30] found that though cues afforded by lexical tones were relatively redundant in terms of Mandarin sentence recognition in clear listening conditions, they were indispensable for the perception of Mandarin sentences in noise. In their study, every word in the sentence was presented with the flat tone (tone 1) in the flat tone (FT) condition and each word in the sentence were assigned a randomly selected tone (from tones 1 to 4) under the random tone (RT) condition. The recognition scores could reach about 95% for Mandarin sentences in FT and RT conditions under clear listening conditions. However, in noisy environments, the performance of the FT and RT conditions both declined significantly to about 70% correct [30]. Similarly, Feng et al. proved that the correctness of sine-wave sentence recognition scores could reach 91.6%, even when sine-wave tone-recognition was only 32.7% correct on average in clear listening conditions, suggesting that the functional contributions of lexical tone to Mandarin sentence recognition were limited in clear listening conditions [28]. In addition, Luo and Fu [59] showed that acoustic information at < 500 Hz contributed strongly to both tone and Mandarin speech recognition under noisy conditions. Furthermore, F0 was proven to play an indispensable role in lexical tone perception under noisy conditions [36, 60], rendering the relative weight of Region 1 larger in noisy than in clear environments.

Third, even in the absence of lexical tone recognition, cues afforded by F0 variation may directly assist speech intelligibility under noisy conditions by focusing the attention of listeners on contextual words and aiding the parsing of continuous speech into meaningful units [30, 61–64]. Mandarin sentence intelligibility decreased if the F0 contours were flattened under noisy conditions, but not in clear conditions [32, 61]. The significance of the dynamic F0 contours in terms of speech intelligibility also applies to non-tonal languages. In one study, speech reception thresholds (SRTs) were recorded for English sentences in which the F0 contours were subjected to various manipulations [62]. Compared to the SRTs when the F0 contours were normal, the SRTs increased when the F0 contours were flattened or inverted.

Regions 2 and 3 were of relatively high importance in terms of Mandarin speech perception in noise. This finding was consistent with those of previous studies suggesting that the middle-frequency region is important for speech recognition [18, 20, 38, 65]. Hopkins and Moore found that the perceptual benefit was greater, compared to envelope cues from any lower or higher ranges, if the temporal envelope in the 397–2041 Hz range was added [24]. Kasturi et al. [18] found that temporal envelope cues from a low-frequency region (300–487 Hz) and middle-frequency region (791–1284 and 1284–2085 Hz) contributed more to vowel recognition than envelope cues from other frequency regions. Vowel perception plays an essential role in Mandarin sentence recognition [31]. Thus, the higher relative weights of the middle-frequency regions (Regions 2 and 3) may be due to the encoding of F_1_ and F_2_, which aids vowel perception for Mandarin under noisy conditions [18, 66]. Moreover, it is believed that formant cues contribute to lexical tone recognition [36, 67, 68], which might further assist Mandarin sentence intelligibility under noisy conditions.

The frequency-weighting functions of the temporal envelope under noisy conditions differed from those derived in clear listening conditions [25]. This is in line with the idea that perception strategies change depending on the environment [9, 38]. It should be noted that the frequency-weighting functions under different noisy conditions also differed from each other. Few differences between frequency-weighting functions for SSN and SAM SSN were observed, suggesting that the effects of the temporal modulation of noise were limited in our study. However, more weights were placed in Region 1 under SAM white noise compared to SAM SSN, indicating that the spectral shape of the noise would impact the frequency-weighting functions of temporal envelope for Mandarin perception. Another reason was that F0 contour might be of higher importance to Mandarin sentence recognition in SAM white noise than in other listening environments [61, 69].

The fact that different frequency regions contribute differently to speech recognition is important in terms of CI development. Due to spectral deterioration, the frequency resolution of CI wearers was poorer than that of those with normal hearing [3, 70–73]. Given that the temporal envelope cues from Region 1 had the highest weight, assigning more channels to the low-frequency region may aid Mandarin sentence recognition under noisy conditions. In addition, extending the frequency range of CIs to include more low-frequency information was demonstrated to aid in Mandarin tone recognition [74]. An increasing amount of evidence suggests that utilizing low-frequency acoustic hearing in bimodal hearing improves lexical tone recognition and Mandarin speech perception under noisy conditions [59, 75–77]. As the frequency-weighting functions change with the listening background, it would be useful if the speech processing strategy of CIs changes automatically with the varying listening background.

However, there are some limitations in our study. First, the test subjects had normal hearing. Although Shannon et al. [16] suggested that the negative effects of holes in different frequency locations were consistent between individuals with normal hearing and CI users, several studies have reported opposite results. Some authors have suggested that the allocation of relative perception weights across frequency regions differs between CI users and listeners with normal hearing [19, 21, 78, 79]. Those suffering from progressive hearing loss increasingly rely on temporal envelope cues rather than TFS to recognize lexical tones [29]. Given the critical role of tone recognition in Mandarin sentence perception in noise, hearing loss will influence the frequency-weighting functions of Chinese-speaking CI users, which is a topic that deserves further study. Second, we used noises to fill the empty holes in our study instead of setting the information in the frequency region to zero to create spectral holes directly [16, 18]; this could not exclude the upward spread of masking of low-frequency filler noise to high-frequency cues. However, high-frequency filler noise will interfere far less in low-frequency speech cues. Third, we only tested Mandarin sentence recognition at a moderate SNR of + 5 dB. Further studies conducted at different SNRs are needed to determine whether the relative weight changes at other SNRs.

## Conclusions

In the presence of noise, temporal envelope cues from a low-frequency region (80–502 Hz) were the most important among the five frequency regions in terms of Mandarin sentence recognition. Compared to the frequency-weighting functions calculated in the clear listening conditions, more functional weights were distributed in the low-frequency region in the noise. Our findings have important clinical implications for optimizing speech-processing strategies for Mandarin Chinese-speaking CI users, particularly in noisy listening environments.

## Supplementary Information

**Additional file 1: Table S1.** The examples of MHINT materials and corresponding translations. **Table S2.** Percent-correct scores in SSN. **Table S3.** Percent-correct scores in SAM SSN. **Table S4.** Percent-correct scores in SAM white noise.

## Data Availability

The datasets used during the current study are not publicly available due to reasons of sensitivity but are available from the corresponding author on reasonable request.

## Abbreviations

ANOVA: Analysis of variance
CI: Cochlear implants
ERB_N_: Equivalent rectangular bandwidth
F0: Fundamental frequency
FT: Flat tone
MHINT: Mandarin version of the hearing in noise test
RAU: Rationalized arcsine units
RT: Random tone
SAM: Sinusoidally amplitude-modulated
SNR: Signal-to-noise ratio
SRT: Speech reception thresholds
SSN: Speech-shaped noise
TFS: Temporal fine structure
